# Characterization and diagnosis of Paget Disease of Bone in historical individuals

**DOI:** 10.3389/fmed.2026.1843305

**Published:** 2026-06-24

**Authors:** Mackenzie A. Dagrosa, Katherine D. Van Schaik

**Affiliations:** 1 Vanderbilt University, Nashville, TN, United States; 2 Vanderbilt University Medical Center, Nashville, TN, United States

**Keywords:** archeological skeletal remains, bone remodeling, differential diagnosis, medieval Europe, osteitis deformans, Paget Disease of Bone (PDB), paleopathology, SQSTM1

## Abstract

Paget Disease of Bone (PDB) is a chronic metabolic disorder characterized by accelerated, disorganized bone remodeling resulting in structural enlargement and mechanical weakness. This review synthesizes 38 historical cases drawn from a three-journal convenience sample restricted to English-language literature; observed geographic patterns should therefore be interpreted with caution, as they may reflect research and publication bias rather than the true paleopathological distribution of the disease. Within our sampled literature, modern clinical cases are declining in prevalence while the archeological record suggests a spike in medieval Northwest Europe characterized by more severe, polyostotic manifestations — though this apparent concentration may partly reflect the geographic focus of the journals searched rather than a true biological pattern. Crucially, a microscopically confirmed case from Byzantine Jordan and a pre-contact case from Ontario demonstrate that PDB existed outside Europe in antiquity, and the apparent rarity of non-European ancient PDB almost certainly reflects under-research of non-European assemblages and non-English-language literature rather than true biological absence. Historically, subjective macroscopic identification of “pumice-like” textures has proven unreliable. Current best-practice protocols require interdisciplinary integration of radiography to identify “blade of grass” lesions and histology to confirm pathognomonic mosaic cement lines. While the SQSTM1 protein was detected with abnormalities in medieval remains, the modern familial *SQSTM1* point mutations were absent — raising the possibility of a distinct ancient pathogenic mechanism. We speculate, as a hypothesis requiring further molecular evidence across broader geographic and temporal samples, that PDB may have undergone a fundamental etiological shift over the past millennium; this interpretation remains provisional and should not be read as a confirmed conclusion.

## 1 Introduction

Paget Disease of Bone (PDB) is a chronic metabolic skeletal disorder and the second most common bone disease globally, surpassed only by osteoporosis. First described by Sir James Paget in 1877 as osteitis deformans, it remains without a confirmed etiology despite nearly 150 years of study. PDB is characterized by excessive bone resorption followed by disorganized bone formation, producing skeletal structures that are enlarged but mechanically weak. In affected individuals, osteoclasts become hyper-nucleated and over-responsive to signaling, leading to the recruitment of osteoblasts that deposit woven rather than organized lamellar bone. Its dramatic decline in modern populations raises an urgent question: if the disease is substantially genetic, why did it affect medieval populations with such disproportionate intensity? This review examines the temporal and geographic distribution of PDB alongside emerging molecular evidence and hypothesizes that the disease may have shifted from an environmentally triggered ancient form to a more genetically determined modern condition.

This review was conducted in August 2025 via targeted searches of the *International Journal of Paleopathology*, *International Journal of Osteoarchaeology*, and the *Journal of the American Association of Biological Anthropology*, supplemented by citation snowballing in Google Scholar and PubMed. Search terms included “Paget disease,” “ancient Paget,” “medieval Paget,” and “ancient osteitis deformans.” Thirty-eight articles describing PDB in specific historical individuals based on macroscopic, radiographic, histological, or molecular evidence were included. General reviews, population-level studies without individual case characterization, and studies lacking primary diagnostic data were excluded. We note that restriction of the initial search to three English-language journals constitutes a convenience sample. The apparent concentration of cases in Northwest Europe may partly reflect geographic and language publication bias toward English-language and European-focused literature, rather than the true global paleopathological distribution of PDB; all geographic conclusions should be interpreted with this limitation in mind.

## 2 The skeletal evidence base

Within our sampled literature, the distribution of reported cases shows a high concentration of confirmed historical PDB in the United Kingdom, particularly in northeast England (1–3). Several findings complicate a strictly northwest European origin theory, however. The Skinner Burial in Ontario (4) documents PDB in the pre-contact Americas, though this represents a single case with inherent limitations, suggesting independent occurrence prior to European contact. A Mesoamerican stone sculpture dated to approximately 715 A.D. has been interpreted as depicting craniofacial changes consistent with “leontiasis ossea” [a descriptive term for craniofacial overgrowth rather than a specific diagnosis (5)] potentially representing PDB in Central America; we note this is an art-historical interpretation rather than a confirmed skeletal diagnosis and should be weighted accordingly. Familial aggregation in Spain (6) further supports a hereditary component in Mediterranean populations. Notably, Kesterke and Judd (7) documented a confirmed PDB case from a Byzantine monastic crypt in Jordan, demonstrating that the disease was present in the Near East in antiquity and directly challenging any strictly Northwest European origin model. Cases span from the Neolithic (8) to the post-medieval period, with a notable concentration of cases within our sample in Medieval European contexts: a pattern that may partly reflect research bias toward English-language, European archeological literature (1, 8–10).

This cluster of cases in medieval Northeast England (1, 9, 10) could point to an infectious disease trigger. Modern histology reveals viral nucleocapsid-like structures in pagetic osteoclasts (11, 12), and one hypothesis is that increased urbanization may have facilitated the spread of a respiratory virus triggering PDB in genetically susceptible individuals (6, 13), though a causal link remains unproven. The paleopathological record reflects modern clinical trends (11) in showing higher prevalence in males and older individuals, consistent with PDB’s nature as a longitudinal disorder of disordered remodeling (1, 10). However, observed male predominance may be partly an artifact of historical burial patterns (14), and monostotic or asymptomatic cases in younger individuals may go undetected in macroscopic screening (10, 15). Geographic clustering in Lancashire (9, 16) and familial aggregation (6) together suggest that neither purely environmental nor purely genetic explanations are sufficient.

## 3 Who makes the diagnosis? Disciplinary perspectives and expertise

Diagnostic authority in PDB research has undergone a clear epistemic shift over the past century. Early case studies by anatomists and physicians (17, 18) relied on gross morphological severity as the primary basis for identification. As clinical radiology matured, authority shifted toward internal visualization: Price (19) and Resnick (20) established that surface changes are secondary to internal remodeling. Contemporary studies (3, 13) reflect a fully interdisciplinary model in which bioarchaeologists supply contextual and population-level data, radiologists provide internal mapping, and geneticists contribute molecular evidence. The result is a hierarchy in which macroscopic assessment serves only as a screening tool, followed by radiographic confirmation and, where resources permit, histological or molecular validation (15, 17, 21) (Table 1).

**TABLE 1:** Disciplinary perspectives and diagnostic authority across key studies in the paleopathological PDB literature.

References	Primary Professional Background	Archeological/study context	Geographic/institutional pattern	Role in diagnostic authority
Mays (10), Mays and Turner-Walker (1)	Bioarchaeologists/physical anthropologists	Wharram Percy; Norton Priory; British population studies	UK; Historic England/Univ. of Southampton	Population screening: established foundational prevalence data for PDB in the UK.
Garnett and Lewis (15)	Bioarchaeologists/clinical researchers	Redhill cemetery population	UK; University of Reading	Methodological critique: identified a 62.5% rejection rate for macroscopic-only diagnoses.
Rogers et al. (21)	Interdisciplinary (paleopathologists & clinicians)	Barton-on Humber population	UK; University of Bristol	Integrative diagnosis: combined visual and radiographic assessment to identify 15 probable cases.
Aaron et al. (29), Bell and Jones (31)	Pathologists/histologists	Wells Cathedral and Barton-on-Humber (Aaron et al.); archaeological pagetic bone samples (Bell and Jones)	UK; University of Leeds and University College London	Microscopic confirmation: confirmed PDB through identification of “mosaic” cement lines.
Stirland (33)	Physical anthropologist	Single individual from Norwich	UK; Norwich Castle Museum	Case study authority: detailed morphological and radiological support for classic PDB presentation.
Shaw et al. (13), Morales-Piga et al. (6)	Interdisciplinary (genetics and clinical medicine)	Norton Priory; modern Spanish familial clusters	European-wide; Univ. of Liverpool/Spanish PDB study group	Etiological evidence: detected the SQSTM1 (p62) protein, with abnormalities, in ancient remains while excluding modern SQSTM1 point mutations (Shaw et al.), and supported a heritable genetic background for modern familial PDB (Morales-Piga et al.).
Kesterke and Judd (7)	Medical specialists/paleopathologists	Byzantine monastic crypt, Jordan	International; Texas A&M University	Geographic breadth: microscopically confirmed PDB outside Northwest Europe, demonstrating ancient disease presence in the Middle East.
Toni and Ceglia (5)	Medical specialists/paleopathologists	Mesoamerican stone sculptures	International; University of Parma and Tufts University	Craniofacial analysis: Interpreted ‘leontiasis ossea’ in sculpture as a possible art-historical expression of PDB; not a confirmed skeletal diagnosis.

## 4 How is the diagnosis made? Methodological approaches

### 4.1 Macroscopic/gross morphological assessment

Visual inspection focuses on gross enlargement, bowing of long bones, “pumice-like” or porous surface texture, and cortical thickening (17, 20, 22, 23). This non-destructive approach enables initial candidate identification in large archeological assemblages (10, 21) but carries substantial risk of overdiagnosis: Garnett and Lewis (15) found that more than half of visually suspected cases were rejected upon radiographic review, with mimics including treponematosis (24) and osteomyelitis (25).

### 4.2 Radiographic analysis

Plain radiography identifies the “blade of grass” lytic wedge, mixed density, and disorganized internal remodeling (20, 26). Consistent imaging of dry bone requires specific kilovoltage parameters (45–55 kV) to compensate for the absence of soft tissue (15). High-resolution digital radiography systems improve diagnostic consistency in archeological specimens. Eight of the 38 reviewed articles explicitly involved radiologists, mirroring the clinical requirement for imaging-defined diagnosis. Garnett and Lewis (15) found that 62.5% of macroscopically suspected cases were rejected after radiographic evaluation, underscoring radiography as the essential confirmatory step.

Cross-sectional imaging enables non-destructive visualization of the “cotton wool” texture in the skull and trabecular coarsening that plain film may miss (7, 27, 28). Elliott et al. (3) demonstrated that radiographic identification of PDB in skeletonized remains can contribute to a biological profile.

### 4.3 Histological/microscopic analysis

Histological methods employ bone biopsy trephines (0.8 cm) or 1 cm × 1 cm mechanically extracted samples (15, 29), dehydrated and embedded in methyl methacrylate without decalcification. Goldner trichrome, toluidine blue, and von Kossa staining under plain and polarized light (15, 29) allow visualization of the pathognomonic mosaic pattern: chaotic, “jigsaw” cement lines produced by rapid, uncoordinated remodeling (17, 18, 30). Backscattered electron (BSE) imaging at 20 keV provides high-resolution mineralization contrast (31). This method is definitive but destructive and requires specialized scanning electron microscopy facilities, limiting its routine applicability (29, 31).

### 4.4 Molecular and chemical analyses

Ancient DNA sequencing at Norton Priory by Shaw et al. (13) produced a nuanced molecular picture. Paleoproteomic analysis successfully identified the SQSTM1 protein (p62), central to the pathological milieu of PDB, in medieval skeletal samples, with Western blotting indicating p62 abnormalities including in dentition. However, targeted DNA sequencing found no evidence of the modern familial *SQSTM1* point mutations at known PDB mutation sites. This distinction is critical: one plausible interpretation of this is that the *SQSTM1* pathway appears to have been involved in ancient PDB, but via a different pathogenic mechanism than in modern disease. High cost, contamination risk, and incompletely defined ancient genetic markers currently limit molecular analysis to specialized research contexts.

### 4.5 Multi-method integration

The most robust diagnoses integrate macroscopic screening with radiographic confirmation and tertiary histological validation (19–21). A clear confidence hierarchy applies: molecular evidence is definitive; radiographic evidence allows confirmation and exclusion of macroscopically suspected cases; and macroscopic evidence alone is insufficient. Garnett and Lewis (15) advocate explicitly for radiographic rejection of visual-only diagnoses to prevent inflation of prevalence statistics.

## 5 PDB in the past: synthesis of findings

### 5.1 Skeletal manifestations in archeological contexts

In historical remains, PDB is most common in the pelvis (67% of cases), followed by the spine, femur, and skull, with the tibia and other long bones less frequently involved (15, 32). Archeological cases predominantly present as polyostotic with extensive skull and limb involvement. Affected bones are characteristically dense, heavy, and deformed (17, 22), with cortical thickening, coarsened trabeculae, and well-defined wavy vascular depressions on the bone surface reflecting pathological hypervascularity (14, 17, 18). Complications include bending deformities, healed fractures, and secondary osteoarthritis (1, 33, 34).

### 5.2 Comparisons with modern clinical PDB

A critical interpretive caveat underlies Table 2, namely that the apparent severity of historical PDB relative to modern cases is substantially a product of sampling bias. Only the most pronounced and structurally robust cases survive taphonomic processes and meet macroscopic screening thresholds, meaning the archeological record systematically over-represents florid, polyostotic disease (14). Monostotic and milder cases, which constitute a significant proportion of modern clinical PDB, are largely invisible in the paleopathological record.

**TABLE 2:** Comparison of modern clinical and historical/archeological manifestations of Paget Disease of Bone.

Feature	Modern clinical cases	Historical/archeological cases
Primary diagnosis	Primarily imaging-based (radiography/scintigraphy) and biochemical markers (11, 28).	Primarily macroscopic screening confirmed by radiology or histology (15, 20, 33).
Prevalence	Declining in the late 20th/early 21st centuries, particularly in former hotspots (10, 14).	Within the sampled literature, a significant spike is recorded during the Medieval period, especially in Northwest England (1, 8–10), although this may represent sampling bias.
Severity	Often less severe or controlled via bisphosphonate treatment (11, 32).	Frequently florid or advanced; untreated cases show extreme deformity and bowing (21, 22).
Distribution	Can be monostotic or polyostotic; often asymptomatic until identified by routine screening (11).	Predominantly polyostotic in the record; bias toward severe cases more readily identifiable in dry bone (15).
Complications	High-output heart failure due to hypervascularity; secondary osteoarthritis (34).	Severe bending deformities, pathological fractures, and secondary osteoarthritis (1, 33).
Genetic profile	Strong association with *SQSTM1* mutations in approximately one-third of familial cases (13).	Absence of modern *SQSTM1* point mutations in analyzed medieval remains; SQSTM1 protein present with abnormalities (13).
Visual texture	“Cotton wool” appearance of the skull on X-ray (11, 28).	“Pumice-like” or porous surface texture on dry bone; coarsened trabeculae (18, 21).

### 5.3 Etiological theories and their support in the archeological record

The etiological landscape of PDB presents a paradox: while the disease reached documented peaks in medieval Northwest England, molecular analysis of Norton Priory remains by Shaw et al. (13) yielded a nuanced finding. The SQSTM1 protein (p62) was successfully identified in medieval skeletal samples with evidence of abnormality, confirming *SQSTM1* pathway involvement. However, targeted DNA sequencing found no evidence of the modern familial *SQSTM1* point mutations, which are the variants that drive contemporary familial cases (13). This suggests that the *SQSTM1* pathway was implicated in ancient PDB via a distinct mechanism, rather than being absent altogether. The investigative weight therefore falls on identifying what alternative genetic or environmental factors activated this pathway in medieval populations. Declines in 20th-century PDB in Lancashire prompted the theory of arsenic exposure from cotton mill wastewater (16), though Boylston and Ogden (9) demonstrated that medieval chronology predates industrialization, undermining that explanation. Modern histology reveals paracrystalline arrays in pagetic osteoclasts resembling paramyxoviruses (11, 12), suggesting zoonotic transmission facilitated by high-density rural environments of the Middle Ages (14). We propose, as a speculative synthesis, that the aggregate evidence is consistent with a multifactorial etiological transition, possibly from an ancient form potentially driven by environmental or infectious triggers activating the *SQSTM1* pathway, to a modern form more directly mediated by heritable *SQSTM1* point mutations. We also acknowledge, however, that this hypothesis rests on limited molecular data from a single site (6, 13).

## 6 Discussion

An open question in PDB paleopathology concerns its origins: within our sample, cases concentrate in Northwest England (1, 2, 10) yet documented cases in pre-contact Ontario (4) and — with appropriate caution regarding the limitations of art-historical evidence — in Mesoamerican sculpture (5) cannot be straightforwardly ascribed to a single regional source. The Kesterke and Judd case from Byzantine Jordan (7) adds further evidence of PDB outside Europe in the pre-modern period. We hypothesize that the apparent European concentration in our sample may reflect a localized environmental stressor, possibly a viral trigger acting on a genetically susceptible population, rather than the geographic origin of the disease itself, though this interpretation is constrained by the geographic and linguistic bias of our search strategy. This distinction has significant implications for how the medieval evidence base should be interpreted and how future archeological surveys should prioritize geographic scope.

A second, methodological controversy concerns diagnostic validity. The 62.5% macroscopic overdiagnosis rate documented by Garnett and Lewis (15) reveals that much of the existing prevalence literature rests on unreliable foundations. The field currently lacks a standardized formal diagnostic scoring system; without one, cross-study comparisons of prevalence remain problematic. Radiographic confirmation by a radiologist should be considered mandatory for any newly reported case, and previously macroscopic-only diagnoses warrant re-evaluation (17, 26).

Current research gaps are substantial. Thousands of potentially pagetic bone fragments remain unanalyzed in museum storage, largely because micro-CT has not been applied routinely to fragmentary assemblages (3). Differential diagnosis protocols need refinement: treponematosis (24) and juvenile rickets (35) both produce overlapping skeletal changes and remain sources of misidentification. Preservation bias, including the preferential survival of dense pagetic bone (14), systematically distorts prevalence estimates in ways that have not yet been formally modeled. Geographic bias in the paleopathological literature is an equally important gap: PDB outside Northwest Europe is significantly under-researched, and the apparent rarity of non-European historical cases almost certainly reflects the concentration of archeological and biomedical research infrastructure rather than true biological distribution. The most promising future directions lie at the intersection of paleopathology and molecular biology. Advances in micro-CT (7, 27, 28) enable high-resolution 3D characterization of trabecular architecture without specimen destruction. Proteomics and ancient DNA sequencing, building on the Norton Priory work (13), offer the means to determine whether the *SQSTM1* pathway differences are consistent across medieval populations and geographic regions, and whether alternative genetic or viral signatures can be identified. Genetic studies of twin cohorts (36) and familial aggregation (6) will be essential to characterizing the modern heritable component against which ancient variation can be compared. Future research must bridge the gap between dry bone morphology and wet-lab molecular data to determine whether PDB is an ancient disease that has biologically evolved or one whose clinical expression has simply changed as its environmental context shifted (13).
